# Development and application of a virus-induced gene silencing protocol for the study of gene function in narrow-leafed lupin

**DOI:** 10.1186/s13007-021-00832-4

**Published:** 2021-12-28

**Authors:** Davide Mancinotti, Maria Cecilia Rodriguez, Karen Michiko Frick, Bjørn Dueholm, Ditte Goldschmidt Jepsen, Niels Agerbirk, Fernando Geu-Flores

**Affiliations:** Section for Plant Biochemistry and Copenhagen Plant Science Centre, Department of Plant and Environmental Sciences, University of Copenhagen, Frederiksberg, Denmark

**Keywords:** Grain legume, VIGS, Pulse, Apple latent spherical virus, *Lupinus angustifolius*, Quinolizidine alkaloids

## Abstract

**Background:**

Lupins are promising protein crops with an increasing amount of genomic and transcriptomic resources. The new resources facilitate the in silico identification of candidate genes controlling important agronomic traits. However, a major bottleneck for lupin research and crop improvement is the in planta characterization of gene function. Here, we present an efficient protocol for virus-induced gene silencing (VIGS) to down-regulate endogenous genes in narrow-leafed lupin (NLL) using the *apple latent spherical virus* (ALSV).

**Results:**

We identified ALSV as an appropriate VIGS vector able to infect NLL without causing a discernible phenotype. We created improved ALSV vectors to allow for efficient cloning of gene fragments into the viral genome and for easier viral propagation via agroinfiltration of *Nicotiana benthamiana*. Using this system, we silenced the visual marker gene *phytoene desaturase* (*PDS*), which resulted in systemic, homogenous silencing as indicated by bleaching of newly produced tissues. Furthermore, by silencing *lysine decarboxylase* (*LaLDC*)—a gene likely to be involved in toxic alkaloid biosynthesis—we demonstrate the applicability of our VIGS method to silence a target gene alone or alongside *PDS* in a ‘PDS co-silencing’ approach. The co-silencing approach allows the visual identification of tissues where silencing is actively occurring, which eases tissue harvesting and downstream analysis, and is useful where the trait under study is not affected by *PDS* silencing. Silencing *LaLDC* resulted in a ~ 61% or ~ 67% decrease in transcript level, depending on whether *LaLDC* was silenced alone or alongside *PDS*. Overall, the silencing of *LaLDC* resulted in reduced alkaloid levels, providing direct evidence of its involvement in alkaloid biosynthesis in NLL.

**Conclusions:**

We provide a rapid and efficient VIGS method for validating gene function in NLL. This will accelerate the research and improvement of this underutilized crop.

**Supplementary Information:**

The online version contains supplementary material available at 10.1186/s13007-021-00832-4.

## Background

Lupins (*Lupinus* spp.) are nitrogen-fixing legumes that can grow on poor soils and accumulate high levels of protein in their seeds (up to ~ 40%) [1]. They have great potential as sustainable, alternative protein crops to meet increasing global demands for dietary protein [2]. Lupins have additional appeal for crop improvement as they have a broad global distribution [3]. Of the four currently cultivated species, three of them are native to the Mediterranean region: narrow-leafed lupin (NLL; *Lupinus angustifolius*), white lupin (*L. albus*) and yellow lupin (*L. luteus*). The fourth cultivated species is the Andean lupin (*L. mutabilis*), which is native to South America and is recognized for its high accumulation of seed oil. Among these four species, NLL is the most widely cultivated and is grown commercially in both Australia and Europe.

As crop plants, lupins are both ancient and modern. While Andean lupin and white lupin were cultivated in antiquity, yellow lupin and NLL have a very young breeding history [1]. One of the major obstacles for the development of lupins as modern crops has been the high level of toxic quinolizidine alkaloids (QAs) that accumulate in the seeds [1]. A breeding milestone was achieved in the first half of the last century, when German and Russian breeders identified the first low-QA individuals of the four cultivated lupin species [4, 5]. For the first time, this allowed the development of ‘sweet’ varieties whose seeds could be consumed without prior extraction of QAs. An intensive Australian breeding program in the 1960s then produced the first successful commercial cultivars by the introgression of basic agronomic traits such as early flowering and resistance to pod shattering [6]. Nowadays, Australia remains the main producer of lupin seed worldwide [2], but in general, lupins are still considered orphan crops. In order for lupins to flourish as alternative protein crops, further breeding efforts are needed in relation to yield, seed composition, resistance to diseases, and even still, grain QA content. It is important to note that while ‘sweet’, low-QA lupins exist, seasonal variation often leads to levels that surpass the safety thresholds established for human and animal consumption [7]. As a consequence, a much better understanding of QA biosynthesis is required in order to develop improved varieties where grain QA levels reliably meet industry thresholds.

Recent years have seen a significant development of genomic and transcriptomic resources that are facilitating the identification of candidate genes controlling important agronomic traits in lupins. For NLL, a draft genome has been assembled [8] and transcriptomic resources exist for both high- and low-QA varieties [9, 10]. For white lupin, genomic and transcriptomic data have been generated for three contrasting genotypes with different degrees of domestication [11]. Further genome sequencing of 39 accessions has recently led to the publication of a white lupin pan-genome [12]. In addition to these comprehensive resources for NLL and white lupin, transcriptomic datasets have been generated for yellow and Andean lupin [13, 14]. Overall, these resources have resulted in the identification of strong candidate genes potentially involved in QA biosynthesis and transcriptional regulation [12, 15, 16], vernalization response [17], and reduced pod shattering [17]; however, these candidates remain to be functionally validated.

It is now apparent that a major bottleneck for lupin research is the characterization of identified gene candidates in planta. Current stable transformation protocols are laborious, time consuming and inefficient with low transformation rates that vary between lupin species and cultivars (up to 2.8%, but generally < 0.45%) [18–21]. The development of mutagenized populations to screen for knockouts in gene targets is likewise cumbersome, and applicability may be limited depending on the chosen genotype. For white lupin, the application of a hairy root transformation protocol is limited since the regeneration of shoots from roots has proved unsuccessful [22, 23]. Finally, in terms of transient assays, there are currently no reports of successful gene expression in NLL using the agroinfiltration method, and NLL might be recalcitrant to this method.

Here, we present a protocol for virus-induced gene silencing (VIGS) to examine the function of endogenous genes in NLL. VIGS is a method used to transiently knock down target gene expression in plants and is employed in a wide range of species [24]. The method exploits the native plant RNA silencing pathway involved in the degradation of viral RNA. When a virus infects a plant cell, the native DICER-like proteins generate small interfering RNAs (siRNAs, 21–24 nucleotides) specific to the viral genome. These siRNAs are integrated into an RNA-induced silencing complex (RISC) that binds to and cleaves intact viral RNA [25]. In VIGS, the viral genome is modified to carry a fragment of a target plant gene. This causes the plant to cleave not only the native viral RNA but also the mRNA of the target gene resulting in post-transcriptional gene silencing [24, 26]. The development of a successful VIGS method relies on the identification of a virus able to infect the plant without inducing severe symptoms. In our protocol, we use the *apple latent spherical virus* (ALSV), which can infect a wide variety of plant species without inducing symptoms in most hosts. ALSV was first characterized as a VIGS vector in 2007 in *Nicotiana benthamiana* [27] and has since displayed a broad host range including *Arabidopsis thaliana,* legumes, and members of the Cucurbitaceae, Solanaceae, and Rosaceae families [28–33].

As an example of the applicability of our VIGS method, we chose to silence a gene involved in the partly elucidated QA biosynthetic pathway, *L. angustifolius lysine decarboxylase* (*LaLDC)*. *LaLDC* was identified by differential transcript screening between high- and low-QA-producing NLL and was most highly expressed in young leaves of the high-QA variety. The gene was found to encode an enzyme able to catalyze the first step of QA biosynthesis—the decarboxylation of l-lysine to form cadaverine—in vitro and when expressed in transgenic *Nicotiana tabacum* and *Arabidopsis* [34]. To date, however, there exists no direct validation of its proposed role in the NLL plant, despite its discovery nearly 10 years ago and the significance of QAs for grain quality.

In summary, we present a rapid and efficient VIGS method to silence genes in NLL. Our work includes the identification of an appropriate virus, optimization of the viral vector and inoculation protocol, and the application of this method to silence the QA biosynthetic gene *LaLDC* to provide direct evidence of its role in QA biosynthesis in NLL. The method can be exploited by other lupin researchers to validate gene function in planta, thus facilitating the improvement of this orphan crop and promoting sustainable agriculture.

## Results

### Modification of ALSV vectors

The ALSV genome is composed of two ssRNA segments—ALSV-RNA1 and ALSV-RNA2—each of which encodes several proteins in the form of one large polyprotein. ALSV-RNA1 (6812 nt) codes for the proteins implicated in viral replication, and ALSV-RNA2 (3384 nt) codes for the movement protein and three capsid proteins (Vp25, Vp20, Vp24). We obtained the ALSV VIGS vector plasmids pEALSR1 and pEALSR2L5R5 containing, respectively, the sequences of ALSV-RNA1 and of ALSV-RNA2L5R5 (3432 nt). The latter is a modified version of ALSV-RNA2 that was previously adapted to carry a plant gene fragment between the movement protein and the Vp25 capsid protein [28]. Traditionally, these non-binary ALSV vectors are first purified from large-scale cultures of *Escherichia coli* and then used to mechanically inoculate *Chenopodium quinoa* for the generation of virions*.* Infected leaves are later used to re-inoculate *C. quinoa* in a virion amplification step. Finally, sap from infected leaves from the second *C. quinoa* batch is used as inoculum for VIGS in target plants [28].

**Fig. 1:**
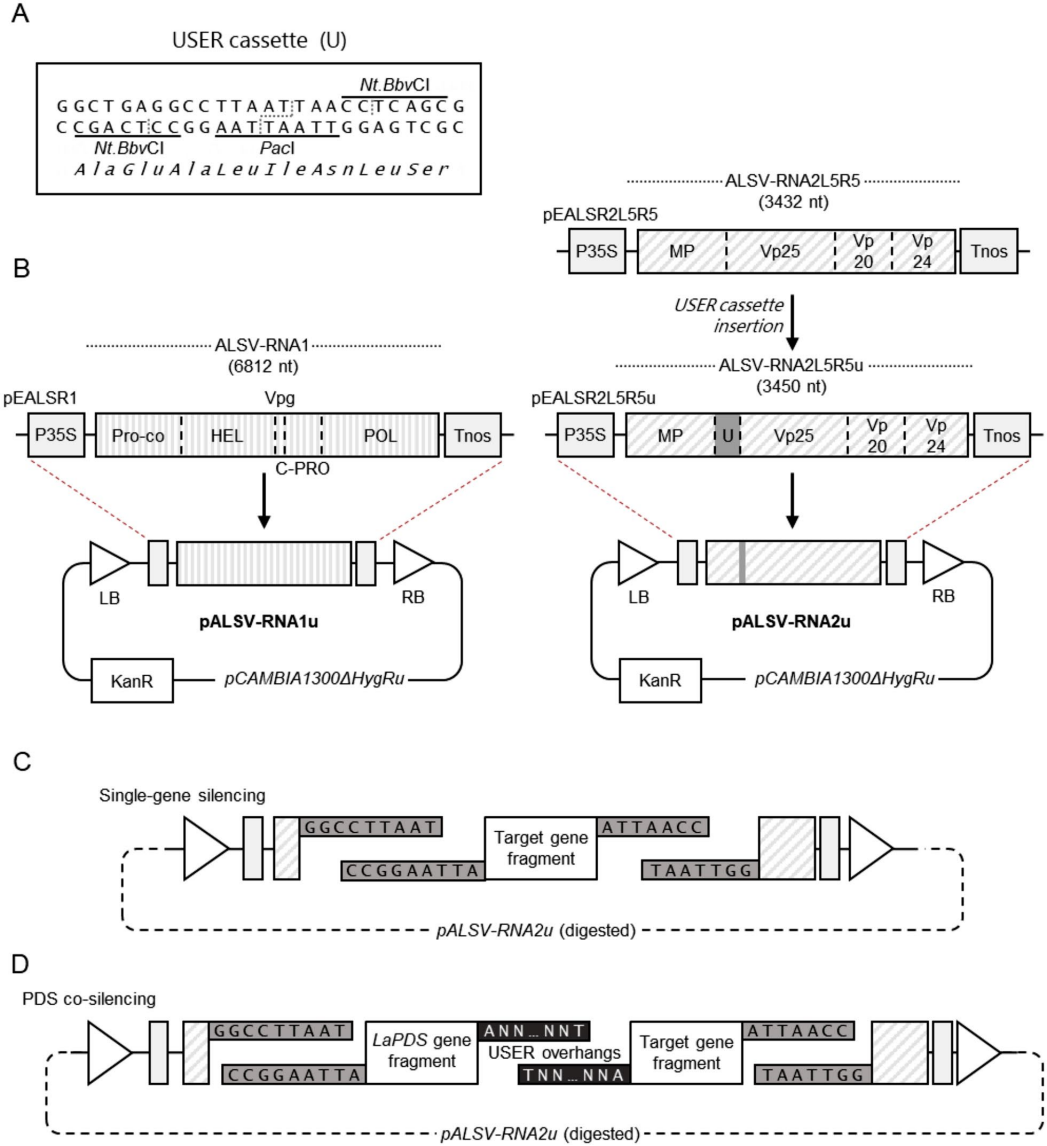
Assembly and functionality of the new ALSV vectors pALSV-RNA1u and pALSV-RNA2u. **A** Sequence of the USER cassette that was introduced into ALSV-RNA2L5R5. The cassette allows for rapid and efficient insertion of one or more target gene fragments. In turn, this allows for VIGS studies with single or multiple gene targets, for example, as part of a PDS co-silencing approach. **B** Cloning strategy for the generation of the new VIGS vectors pALSV-RNA1u and pALSV-RNA2u. The USER cassette detailed above was introduced into ALSV-RNA2L5R5 between MP and VP25 to create pEALSR2L5R5u. Then, the full ALSV expression cassettes from pALSR1 and pALSR2L5R5u were sub-cloned into the binary vector pCAMBIA1300ΔHygRu. **C** Schematic diagram of the USER cloning of a single target gene fragment as part of a single-gene silencing approach. **D** Schematic diagram of the USER fusion reaction where two fragments—an *LaPDS* fragment and a target gene fragment—are simultaneously fused and cloned as part of a PDS co-silencing approach. Abbreviations: P35S, 35S promoter from the *cauliflower mosaic virus* (CaMV); PRO-co, protease cofactor; HEL, NTP-binding helicase; Vpg, viral protein genome-linked; C-PRO, cysteine protease; POL, RNA-dependent RNA polymerase; MP, movement protein; VP25, VP20 and VP24, capsid proteins; Tnos, nopaline synthase terminator from *A. tumefaciens*; LB and RB, T-DNA left and right borders; KanR, kanamycin kinase (resistance gene). Dashed boxes represent the ALSV-RNA1 and ALSV-RNA2 polyprotein-coding sequences. Solid, grey boxes represent the USER cassette and associated overhangs. The solid, black boxes represent custom, USER-compatible overhangs

To improve the earlier method, we generated two new ALSV vectors compatible with ligation-independent cloning and agroinfiltration: pALSV-RNA1u and pALSV-RNA2u (Fig. 1). For this, we first introduced a USER cassette [35] (Fig. 1A) into ALSV-RNA2L5R5 to give ALSV-RNA2L5R5u (3450 nt). We then cloned ALSV-RNA1 and the newly generated ALSV-RNA2L5R5u into the binary vector pCAMBIA1300ΔHygRu [36], thus obtaining pALSV-RNA1u and pALSV-RNA2u, respectively (Fig. 1B). The new ALSV vectors allow for easier viral propagation via agroinfiltration in *N. benthamiana* and offer compatibility with the USER cloning system, which allows for the simultaneous cloning of single or multiple target gene fragments into pALSV-RNA2u (Fig. 1C and D). When cloning target gene fragments into pALSV-RNA2u, the fragment(s) must not generate premature stop codons or alter the reading frame of the viral polyprotein.

### ALSV as a suitable VIGS vector in NLL

**Fig. 2:**
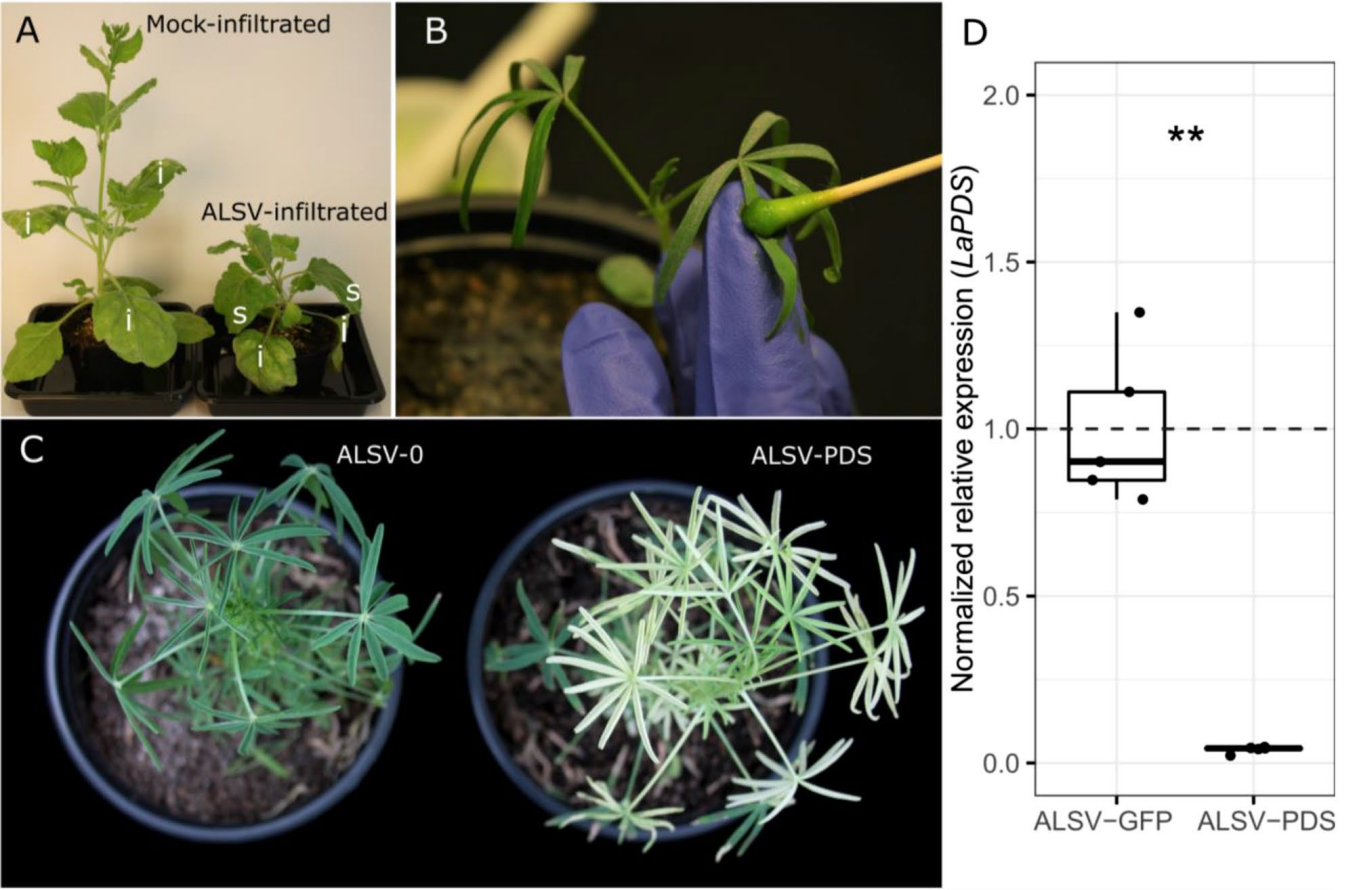
Key steps of the ALSV-based VIGS protocol in NLL and its application for the silencing of *LaPDS*. **A** Virion production via agroinfiltration in *N. benthamiana*. The image shows representative plants 10–15 days post-agroinfiltration with either an empty *Agrobacterium* strain (mock-infiltrated) or a mixture of strains carrying pALSV-RNA1u, pALSV-RNA2u with or without target gene fragment, and pEAQ-HT-DEST1 (for the expression of P19) (ALSV-infiltrated). Only the ALSV-infiltrated plant shows viral symptoms such as stunting and necrosis on leaves and petioles. i, infiltrated leaves; s, systemic leaves. **B** Rub-inoculation of young NLL plants with *N. benthamiana* leaf sap from ALSV-infiltrated plants. **C** Effective silencing of *LaPDS* in NLL. The image shows representative NLL plants inoculated with ALSV without a target gene fragment (ALSV-0) or with a fragment of *LaPDS* (ALSV-PDS). ALSV-PDS-infected plants typically develop bleaching about 5 weeks after inoculation. **D** Normalized relative expression of *LaPDS* in plants infected with ALSV carrying a fragment of GFP (ALSV-GFP; control) or ALSV-PDS. Box plots represent data for five biological replicates. Whiskers represent values within 1.5 times the interquartile range over or under the 75th and 25th percentiles, respectively. Dots represent the individual data points, the dashed line represents the mean of ALSV-GFP, and the asterisks indicate a significant difference (two-sided Wilcoxon test; *P* ≤ 0.01)

We tested our modified VIGS vectors in NLL by downregulating *phytoene desaturase* (*PDS*), a gene involved in the biosynthesis of carotenoids in photosynthetic tissues [37]. *PDS* is a common visual marker for VIGS systems and its silencing results in a bleached phenotype [24]. We identified the NLL gene with greatest homology to the *PDS1* gene from soybean (M64704) [28], designated it *LaPDS,* and cloned a 181-bp cds fragment into pALSV-RNA2u. As a negative control, we cloned a 178-bp cds fragment of GFP into pALSV-RNA2u. Since NLL appears recalcitrant to transient gene expression via agroinfiltration, we first propagated the two different ALSV versions (ALSV-PDS and ALSV-GFP, respectively) in the highly susceptible plant *N. benthamiana*, including co-infiltration with a strain carrying the known silencing suppressor P19 [38]. Viral propagation in *N. benthamiana* resulted in a characteristic stunting of plant growth, bending of stems and petioles, and necrotic lesions on infiltrated and systemic leaves (Fig. 2A). After confirming the presence of the unabridged ALSV variants in systemic leaves by RT-PCR (Additional file 1: Fig. S1), we rub-inoculated 1-week old NLL with sap from the systemic *N. benthamiana* leaves. At about 5 weeks post-inoculation, we observed bleaching of leaves in the NLL plants infected with ALSV-PDS (Fig. 2B, C). The bleaching was only observed on new growth, where it appeared homogeneous and persisted over at least 3 weeks. Across 5 replicate experiments, we observed a bleaching frequency of 40–95% (percentage of inoculated plants that showed bleaching) recorded 2–3 weeks after the first appearance of discoloration in the batch of infected plants. We also observed that the infection of NLL with ALSV-GFP or with empty ALSV vector (ALSV-0) did not result in any visible symptoms in comparison to uninfected plants (not shown). We measured the extent of downregulation of *LaPDS* in bleached tissues using qPCR and found a very strong silencing of ~ 96% compared to ALSV-GFP control (Fig. 2D). Altogether, these results indicate that our VIGS vectors and inoculation protocol can induce a marked and homogenous gene silencing in NLL leaves.

### Applying the ALSV system to validate the function of LaLDC

To test the potential of our VIGS method to validate gene function in lupins, we silenced a gene regarded as involved in the first step of QA biosynthesis, *LaLDC* [34]. We amplified a 196-bp *LaLDC* cds fragment from NLL, cloned it into pALSV-RNA2u, and produced the corresponding virions in *N. benthamiana* (ALSV-LDC). We then inoculated the virions onto NLL, separately including ALSV-GFP as a negative control and ALSV-PDS as a visual marker to indicate the onset of silencing. Plants infected with ALSV-PDS started bleaching at 35 days post-inoculation. Twenty days later, we harvested leaves from 8 plants selected at random among those infected with ALSV-GFP and ALSV-LDC, respectively.

**Fig. 3:**
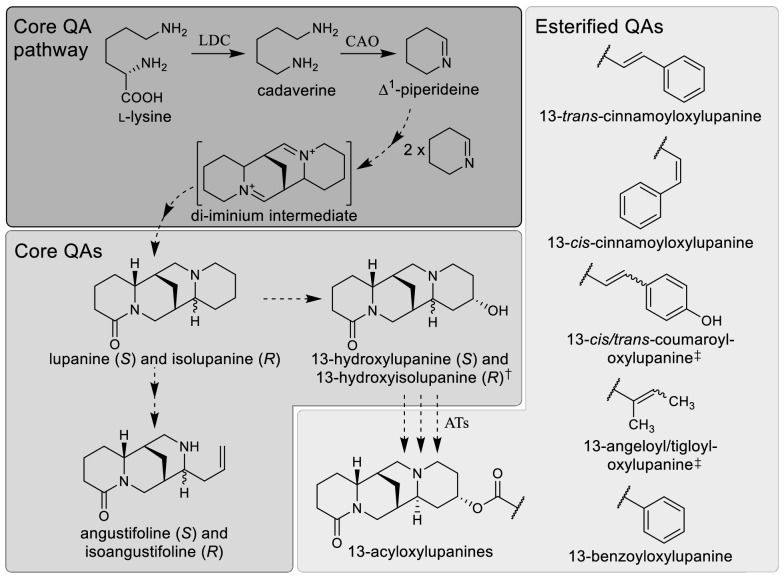
QA biosynthetic pathway in NLL and classification of QAs into core and esterified QAs. QAs are synthesized from l-lysine, which is decarboxylated by LDC to yield cadaverine. In turn, cadaverine is oxidatively deaminated to ∆^1^-piperideine by CAO. Three ∆^1^-piperideine molecules are then joined to form a four-ring, putative di-iminium intermediate from which all core QAs are thought to be derived [40]. The simplest QAs that accumulate in NLL are lupanine and isolupanine. These two QAs can be converted to angustifoline and isoangustifoline via ring opening with loss of one carbon or to 13-hydroxylupanine and 13-hydroxyisolupanine via hydroxylation. Altogether, these six compounds constitute the main core QAs in NLL. Additionally, acyltransferases (ATs) can conjugate 13-hydroxylupanine to various acyl-CoA derivatives to form an array of esterified QAs. Abbreviations: LDC, lysine decarboxylase; CAO, copper amine oxidase; ATs, 13-hydroxylupanine *O*-acyltransferases. Plain and dashed arrows indicate, respectively, characterized and predicted steps. The (*R*) and (*S*) labels refer to the absolute configuration at the carbon atom marked with the wavy bond. ^†^These isomers were analyzed together as a single compound. ^‡^Only one of these isomer pairs accumulates in the leaves of NLL cv. Oskar; we did not determine which one

**Fig. 4:**
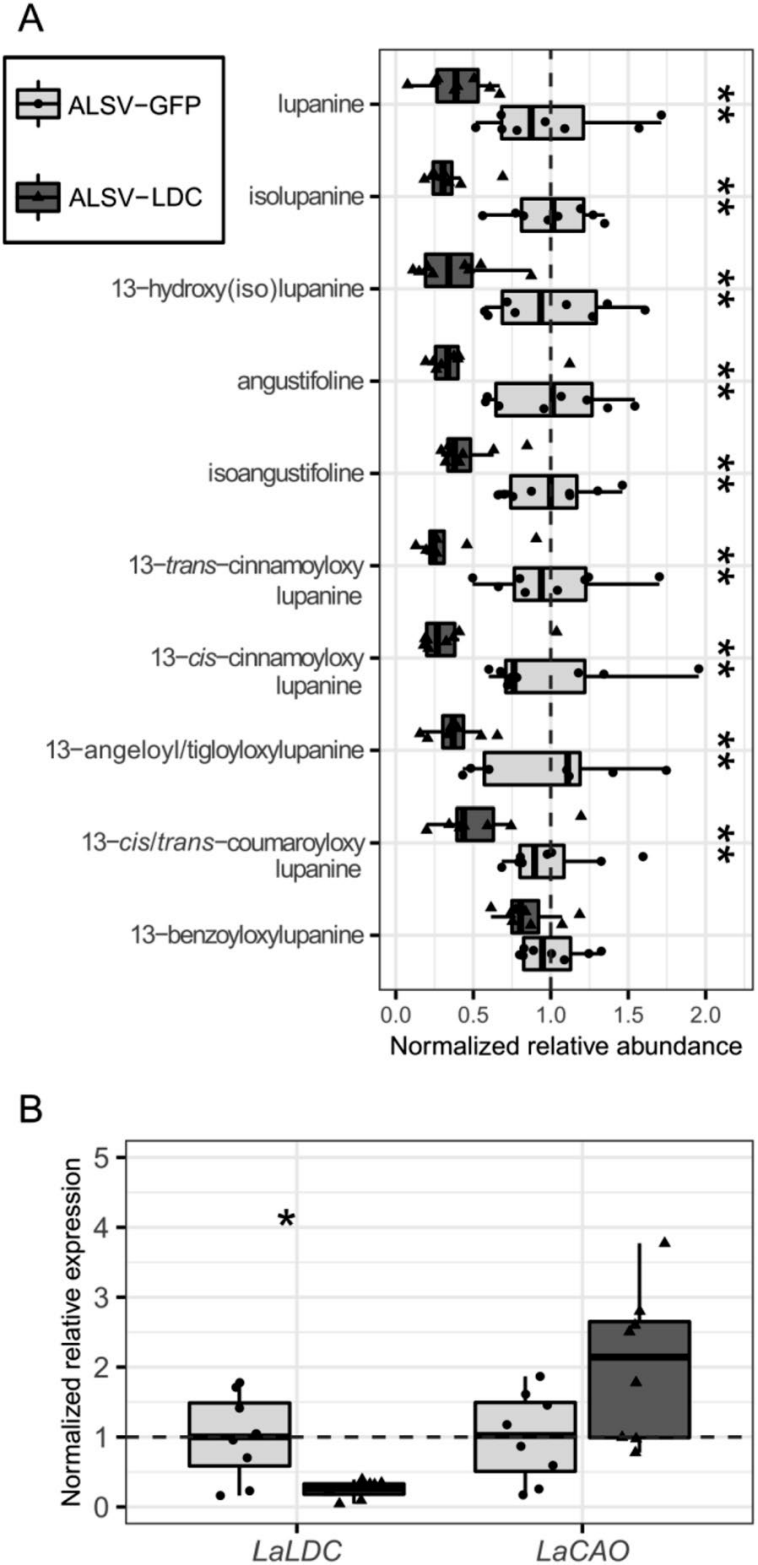
Silencing of *LaLDC* in NLL leaves using ALSV. **A** Normalized relative abundance of QAs in plants infected with ALSV-GFP or ALSV-LDC. **B** Normalized relative expression of *LaLDC* and *LaCAO* in plants infected with ALSV-GFP or ALSV-LDC. Box plots represent data for eight biological replicates. Whiskers represent values within 1.5 times the interquartile range over or under the 75th and 25th percentiles, respectively. Dots and triangles represent the individual data points, dashed lines represent the mean of ALSV-GFP, and significant differences are represented by one or two asterisks (two-sided Wilcoxon test; *P* ≤ 0.05 or *P* ≤ 0.01, respectively)

We then used LC–MS to compare the levels of the 10 most abundant QAs found in the harvested leaves. These QAs can be categorized as either ‘core’ or ‘esterified’, with core QAs representing unmodified or minimally modified QA backbones (e.g., lupanine) and esterified QAs representing larger modifications resulting from conjugation to CoA-activated acids (e.g., 13-*trans*-cinnamoyloxylupanine) (Fig. 3) [39]. There was a statistically significant difference in the relative amount of QAs between plants infected with ALSV-GFP or ALSV-LDC (MANOVA; *P* = 0.035). For plants infected with ALSV-LDC, all identified QAs except for the esterified QA 13-benzyloyloxylupanine were significantly reduced by 45–60% compared to control plants (Fig. 4A). This reduction in QA content was associated with a ~ 61% reduction in *LaLDC* transcript level, as revealed by qPCR. We also investigated how the silencing of *LaLDC* may affect the regulation of QA biosynthesis. While most of the QA biosynthetic pathway is unknown, a strong candidate for the second enzyme in the pathway has been identified as *L. angustifolius* copper amine oxidase (LaCAO) [10]. We measured the transcript level of *LaCAO* and, while average levels were higher in plants infected with ALSV-LDC compared to control plants, the difference was not significant (Fig. 4B). Overall, these results provide direct evidence that *LaLDC* is a major gene involved in QA biosynthesis in NLL.

Upon carrying out a replicate experiment however, it became apparent that the use of ALSV-PDS was not a completely reliable indicator of the onset of silencing for the different ALSV constructs. In this replicate experiment, we observed a similar decrease in the levels of most QAs in plants infected with ALSV-LDC compared to ALSV-GFP, but the observed downregulation of *LaLDC* was not significant (not shown). The fact that we did observe the same chemotype suggests that silencing of *LaLDC* occurred at an earlier time point and then subsided. Indeed, due to their transient nature, VIGS systems in plants are often reported to be variable concerning the timing and extent of silencing [24, 41, 42]. Therefore, we decided to test a ‘PDS co-silencing approach’ where we silenced our visual marker *LaPDS* alongside the target gene *LaLDC* using a single construct. We envisaged that the visible silencing of *LaPDS* would occur concurrently with the silencing of *LaLDC* and would aid the timely harvest of silenced leaf material.

### Validating the function of LaLDC using a ‘PDS co-silencing’ approach

We constructed an ALSV variant carrying a fusion of the previously tested *LaPDS* and *LaLDC* fragments (ALSV-PDS-LDC). As negative control, we constructed an analogous ALSV variant where the mentioned *LaLDC* fragment was replaced by the previously tested *GFP* fragment (ALSV-PDS-GFP). When testing these ALSV variants in NLL, we also inoculated plants with our original visual marker ALSV-PDS as well as with ALSV-GFP, as previously described.

At 54 days after inoculation, we observed bleaching in ~ 57% of the plants infected with ALSV-PDS, whereas we observed similar effects in ~ 12% of the plants infected with ALSV-PDS-GFP (7 plants) or ALSV-PDS-LDC (10 plants). To assess whether the PDS co-silencing approach was at all suitable for the study of QA biosynthesis, we first verified that the accumulation of QAs was not disrupted in *LaPDS*-silenced leaves. Indeed, the levels of QAs in the bleached leaves of ALSV-PDS plants were similar or moderately higher than the levels in the green leaves of ALSV-GFP plants, with the exception of 13-*trans*-cinnamoyloxylupanine, which was ~ 50% lower (Additional file 1: Fig. S2).

**Fig. 5:**
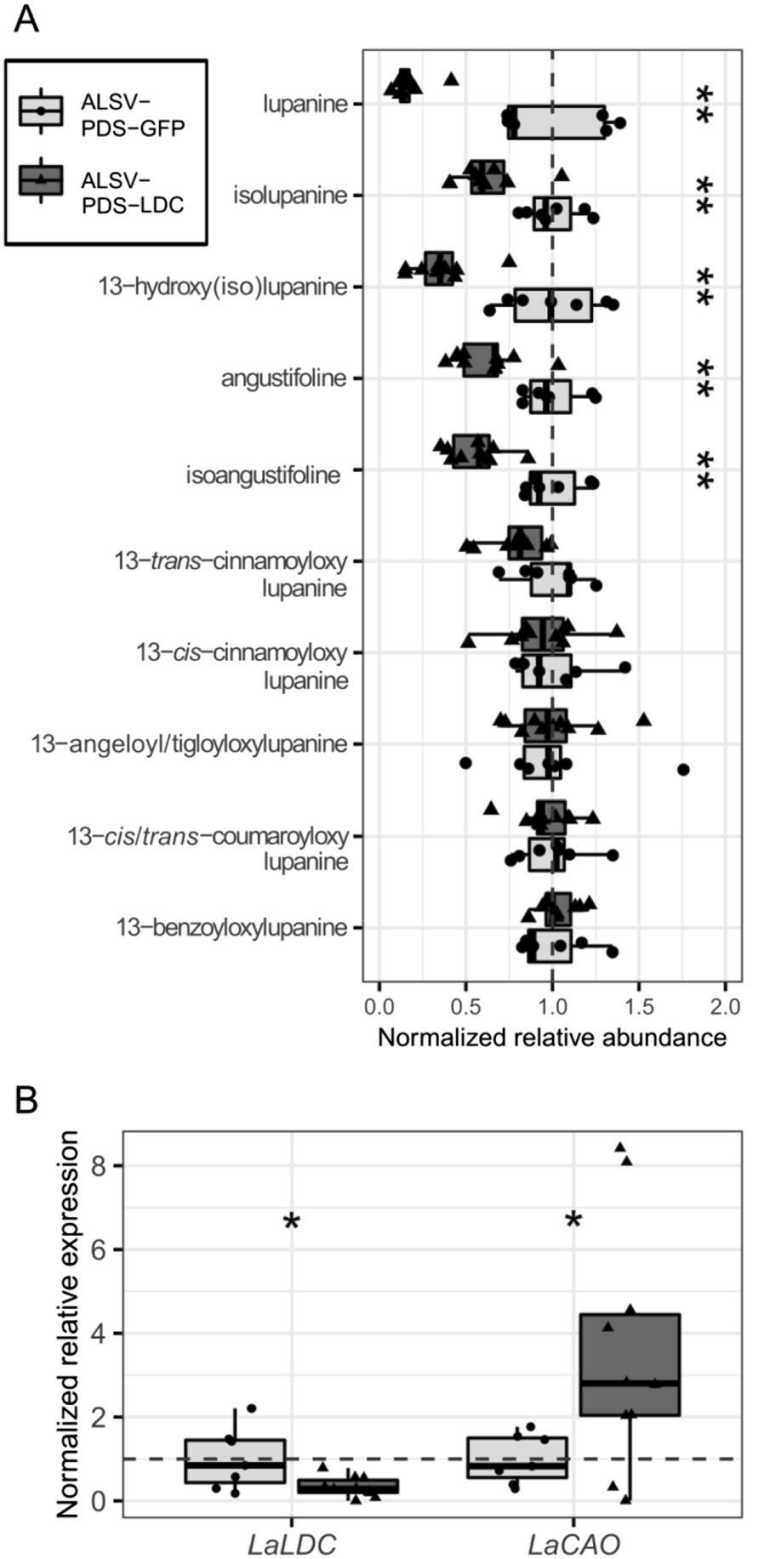
Simultaneous silencing of *LaLDC* and *LaPDS* in NLL leaves using a PDS co-silencing approach. **A** Normalized relative abundance of QAs in bleached leaves of plants infected with ALSV-PDS-GFP or ALSV-PDS-LDC. **B** Normalized relative expression of *LaLDC* and *LaCAO* in plants infected with ALSV-PDS-GFP or ALSV-PDS-LDC. Box plots represent data for 7 and 10 biological replicates for plants infected with ALSV-PDS-GFP and ALSV-PDS-LDC, respectively. Whiskers represent values within 1.5 times the interquartile range over or under the 75th and 25th percentiles, respectively. Dots and triangles represent individual data points, dashed lines represent the mean of ALSV-PDS-GFP, and significant differences are represented by one or two asterisks (two-sided Wilcoxon test; *P* ≤ 0.05 or *P* ≤ 0.01, respectively)

We then analyzed QAs in bleached leaves from plants infected with ALSV-PDS-LDC and compared them to bleached leaves from control plants infected with ALSV-PDS-GFP. There was a statistically significant difference in the relative amount of QAs between plants infected with ALSV-PDS-GFP and ALSV-PDS-LDC (MANOVA; *P* = 0.004). Specifically, we observed a significant decrease of 37–83% in the levels of all core QAs, particularly lupanine, in the ALSV-PDS-LDC samples compared to the controls. The esterified QAs, instead, were unaffected (Fig. 5A). The altered QA levels were associated with a ~ 67% decrease in *LaLDC* transcript level and a ~ 252% increase in *LaCAO* transcript level (both significant, Fig. 5B). We measured the expression of *LaPDS* in bleached leaves from plants infected with ALSV-PDS and ALSV-PDS-GFP in comparison to those infected with ALSV-GFP and found that *LaPDS* silencing was similar in the PDS co-silencing approach as when *LaPDS* was silenced alone (Additional file 1: Fig. S3).

Despite the differences in how the QAs were affected when silencing *LaLDC* alone or when silencing it via the PDS co-silencing approach, we consistently observed a decrease in the measured core QAs with both strategies. We therefore conclude that PDS co-silencing is an appropriate method to functionally characterize genes involved in QA biosynthesis. Additionally, we were able to reproduce the results (both chemotype and transcript levels) and conclude that the new approach is more reproducible than silencing the target gene alone (data not shown).

## Discussion

The functional characterization of genes in lupins has long been a challenge due to the lack of efficient tools for the selective manipulation of gene expression. In this work we have identified ALSV as a suitable VIGS vector for NLL and have developed a rapid and efficient VIGS protocol for validating gene function in this promising crop. Each experiment can be conducted in 2–3 months from start to finish, given that virus propagation in *N. benthamiana* takes 10–15 days post-agroinfiltration and silencing in NLL takes 5–8 weeks post-inoculation. ALSV is an ideal VIGS vector as it does not cause a discernible phenotype in NLL. Furthermore, ALSV does not seem to be transmitted easily from plant to plant, and moderately high temperatures (37 °C) can disable the movement of virus within the plant, thus helping with virus containment and control [43, 44]. In a 2015 report, a VIGS method based on the *peanut stunt virus* was used to silence the common marker gene *PDS* in white lupin [45]. In this case, however, *PDS* silencing resulted only in weak bleaching that appeared restricted to leaf veins, and the method has not been used to functionally validate other lupin genes. With our protocol, ALSV induces a marked gene silencing in NLL leaves, as evidenced by a homogeneous and prolonged bleaching of tissues. Considering that ALSV has proved to be an efficient VIGS vector in several legume species [28], our VIGS protocol is an ideal starting point for testing ALSV-mediated gene silencing in the three other cultivated lupin species.

Furthermore, we have demonstrated the applicability of our VIGS method to silence a target gene alone or in a ‘PDS co-silencing’ approach. We developed the PDS co-silencing approach to improve reproducibility and decrease the time, effort, and costs associated with the VIGS experiments. In general, the effectiveness of VIGS systems is often reported as variable with the timing and levels of silencing varying between batches of plants, individual plants, or even patches of cells from a single tissue type [24, 41, 42]. This makes it difficult to determine the exact tissue and time point at which silencing of the target gene is occurring when using the traditional protocols. This is particularly relevant for viruses like ALSV, which do not cause visible symptoms in the host plant. A similar PDS co-silencing approach has been employed very recently in two other VIGS systems in *Antirrhinum majus* [46] and *Catharanthus roseus* [41]. In the case of *C. roseus*, the PDS co-silencing strategy was key to discovering a gene that resisted identification using the traditional, single-target VIGS method [41]. Apart from improving reproducibility, this approach allows decreasing the number of biological replicates per VIGS construct, thus greatly reducing the experimental input associated with harvesting, extraction, and downstream analysis (e.g., qPCR and LC–MS). However, the single-gene strategy may still be necessary when the biological traits under study are too perturbed by the silencing of *PDS*, which affects chloroplast development [47]. In the mentioned *C. roseus* study, the accumulation of alkaloids was not considerably altered upon *PDS* silencing, and the phenotypes of the silenced biosynthetic genes could still be observed. We noted a similarly small effect of *PDS* silencing on alkaloid biosynthesis in NLL and therefore conclude that our method can be used for validating gene function in QA biosynthesis. Nevertheless, we have observed that bleached leaves undergo early senescence (data not shown), and thus, when using the *PDS* co-silencing strategy, we recommend harvesting of tissue soon after the onset of bleaching.

The PDS co-silencing approach also demonstrates the ability of our ALSV-based VIGS system to silence more than one gene using a single viral construct. This provides the opportunity to silence potentially redundant genes simultaneously, where a phenotype might not be observed by silencing only one of the genes. It has been reported that the size of the fragments inserted into the viral genome can have an effect on the efficiency of silencing, with vectors with larger inserts being more susceptible to losing their inserts or limiting systemic movement of the virus, thus leading to less efficient silencing [48, 49]. Unexpectedly, we observed a similar extent of silencing of both *LaPDS* and *LaLDC* when the respective fragment was silenced alone as a ~ 180 bp insert or when fused with another gene fragment giving a ~ 370 bp insert (Figs. 4, 5, and Additional file 1: Fig. S3). However, the bleaching frequency among the plants infected with the ALSV constructs for co-silencing was considerably lower than among those infected with ALSV-PDS. Therefore, we suggest inoculating at least 50 plants per construct for co-silencing. Compared to the single-gene strategy, the increased efforts during plant inoculation are more than compensated by the decreased workload during harvesting and downstream analysis.

Finally, we provide crucial evidence that *LaLDC* is indeed involved in QA biosynthesis in NLL. Even though *LaLDC* has been regarded as having a role in QA biosynthesis, direct evidence of its involvement has been missing. It is known that sweet NLL cultivars harboring the low-alkaloid locus *iucundus* have lower levels of *LaLDC* expression compared to high-QA cultivars [34]. However, the levels of *LaCAO* are also lower in these sweet cultivars [10, 16], making it impossible to attribute the low-alkaloid phenotype solely to the low expression of *LaLDC*. Furthermore, genetic mapping in conjunction with the NLL genome draft has excluded the possibility that *iucundus* and *LaLDC* are the same gene, since they are placed on different linkage groups [8]. In our experiments, we were able to downregulate *LaLDC* without negatively affecting *LaCAO*, and this led to markedly reduced levels of most QAs (Figs. 4 and 5). Interestingly, the downregulation of *LaLDC* led to a significant upregulation of *LaCAO* in our PDS co-silencing approach, suggesting that the reduced QAs levels activated a yet uncharacterized feedback regulation system. As more research is carried out to elucidate the QA pathway in lupins, we envision that our VIGS system will become a valuable workhorse for uncovering novel pathway steps and assigning genes and enzymes to each step.

## Conclusions

We have developed an efficient system for VIGS in NLL that results in effective and homogenous gene silencing. Furthermore, we have demonstrated the applicability of this method to validate gene function via single-gene silencing or using a ‘PDS co-silencing’ approach to reduce time, cost, and effort. We have also demonstrated that silencing of *LaLDC* leads to a marked decrease in QA levels, providing the first direct evidence of the role of this gene in QA biosynthesis in NLL.

## Methods

### Plant growth conditions

Seeds of *Lupinus angustifolius* cv. Oskar (a high-QA cultivar obtained from Hodowla Roślin Smolice, Poland) were soaked for 2 h in a suspension of *Rhizobium* bacteria (RADICIN-Lupin, JOST GmbH, Iserlohn, Germany) and sown in 16 cm-wide, 20 cm-deep pots at a density of five seeds per pot. The potting medium was prepared by mixing equal parts of sterilized, peat-based potting soil, sand, and 0–4 mm expanded clay pebbles. The plants were grown under greenhouse conditions at day/night temperatures of 21/18 °C, a light/dark photoperiod of 16/8 h, and at 60% relative humidity. Artificial light was supplied to keep the PPFD above 150 µmol/m^2^/s during the day.

*Nicotiana benthamiana* seeds were sown directly on peat-based potting soil, grown under the conditions described above, and individually transplanted to 10 cm-wide, 8 cm-deep pots at an age of 3 weeks.

### Modification of ALSV constructs

First, a USER cassette consisting of the two phosphorylated oligos ALSV2_USER(+) and ALSV2_USER(−) (see sequences in Additional file 1: Table S1) was inserted between the *Xho*I and *Bam*HI restriction sites of pEALSR2L5R5 [28] to generate the plasmid pEALSR2L5R5u. Second, the hygromycin resistance gene and the CaMV 35S promoter were removed from the plasmid pCAMBIA130035Su by cutting and re-ligating sequentially with *Xho*I and *Pst*I, which yielded the plasmid pCAMBIA1300ΔHygRu. Third, the ALSV-RNA1 and ALSV-RNA2L5R5u viral genomes, together with their promoter and terminator regions, were sub-cloned into pCAMBIA1300ΔHygRu. For this purpose, the ALSV-RNA1 expression cassette was amplified from pEALSR1 as three separate fragments using primer pairs ALSV_35S_Fw/ALSV1_Frag1_Rv, ALSV1_Frag2_Fw/ALSV1_Frag2_Rv, and ALSV1_Frag3_Fw/ALSV_Tnos_Rv (Additional file 1: Table S1). Similarly, the ALSV-RNA2L5R5u expression cassette was amplified from pEALSR2L5R5u as two separate fragments using primer pairs ALSV_35S_Fw/ALSV2_Frag1_Rv and ALSV2_Frag2_Fw/ALSV_Tnos_Rv (Additional file 1: Table S1). The respective PCR fragments were simultaneously fused and cloned into pCAMBIA1300ΔHygRu via USER fusion [35] giving pALSV-RNA1u and pALSV-RNA2u (Fig. 1).

### Cloning gene fragments into pALSV-RNA2u

We designed pALSV-RNA2u to be able to carry a gene fragment between the movement protein and the Vp25 capsid protein upon USER cloning (Fig. 1). Target gene fragments must be cloned into pALSV-RNA2u without generating premature stop codons or altering the reading frame of the viral polyprotein. The fragments were amplified from leaf cDNA [10] with USER-compatible primers (Additional file 1: Table S1). Cloning reactions were composed of 10 µL of purified PCR fragment(s), 1 µL of *Pac*I/*Nt.Bbv*CI-digested pALSV-RNA2u vector (ca. 50 ng), and 1 U of USER enzyme mix (New England Biolabs). The reaction was incubated at 37 °C for 20 min and transformed into *E. coli* without ligation*.* Correct insertions were verified by Sanger sequencing and the new constructs were transformed into *Agrobacterium* strain AGL-1. For the single-gene silencing strategy, individual fragments of approximately 180 bp from *LaPDS*, *LaLDC*, and *GFP* were used. For the PDS co-silencing strategy, primers were designed to amplify and fuse together the same gene fragments used for the single-gene strategy to give a total insert size of approximately 370 bp. The coding sequences of *LaPDS*, *LaLDC*, and *GFP* are shown in Additional file 1: Sequences S1, S2 and S4, respectively, and the VIGS fragments are highlighted therein.

### Propagation of ALSV in *N. benthamiana*

*Agrobacterium* strains separately harboring the pALSV-RNA2u plasmids with respective target inserts, the pALSV-RNA1u plasmid, and pEAQ-HT-DEST1 (for the expression of RNA silencing suppressor P19 from the *tomato bushy stunt virus*) [50] were cultured at 28 °C with shaking until an OD_600_ of approximately 3 was reached. Bacterial pellets were collected by centrifugation and resuspended in water to an OD_600_ of 1. The resuspensions harboring pALSV-RNA2u with a target insert were mixed in equal volumes with the ones harboring pALSV-RNA1u and pEAQ-HT-DEST1. Strain mixtures were infiltrated into the abaxial side of three young leaves of 4-week-old *N. benthamiana* plants using a 3 mL needleless syringe. Approximately 10–15 days post-agroinfiltration, *N. benthamiana* plants started to show viral symptoms, characterized by stunting, leaf distortion and necrosis (Fig. 2A). The 2–3 leaves immediately above the youngest infiltrated leaf were collected, flash-frozen in liquid nitrogen, quickly ground in a mortar to a very coarse powder, and stored at − 80 °C. To confirm the presence of ALSV in the systemic *N. benthamiana* leaves we harvested, we extracted RNA and performed RT-PCR using the primers pALSV-RNA2u_Fw/pALSV-RNA2u_Rv, which flank the USER cassette (Additional file 1: Fig. S1, Table S1).

### Inoculation of NLL with ALSV

The inoculation of NLL with ALSV was carried out using *N. benthamiana* leaf sap inoculum prepared on the same day. To prepare the inoculum, 1 g of frozen leaf powder was homogenized in 5 mL of inoculation buffer (100 mM NaCl, 5 mM MgCl_2_, 100 mM Tris–HCl, pH 7.8) and kept on ice until use. NLL seedlings with two true leaves (approximately 1 week after sowing) were mechanically inoculated by first generously dusting them with carborundum—an abrasive powder—and then rubbing the adaxial side of leaves and cotyledons with cotton buds dipped in the *N. benthamiana* leaf sap inoculum. Once the plants inoculated with ALSV-PDS, ALSV-PDS-GFP or ALSV-PDS-LDC started to show bleaching (at approximately 5 weeks post-inoculation), young leaves at similar developmental stages (bleached leaves in the case of *LaPDS*-silenced plants) were flash frozen in liquid nitrogen, pulverized with a steel ball mill homogenizer and stored at − 80 °C until further analysis.

### Analysis of gene expression by qPCR

RNA was extracted from 100 mg of NLL leaf tissue using Spectrum Plant Total RNA kit (Sigma-Aldrich) including on-column DNase I digestion (Sigma-Aldrich). cDNA from 1 µg RNA was then synthesized using the iScript cDNA Synthesis Kit (Bio-Rad). For quantification of silenced gene targets, qPCR primers (Additional file 1: Table S1) were designed outside the region cloned into the pALSV-RNA2u vector (Additional file 1: Sequences S1–S3). A ubiquitin gene [51] was used as a reference gene for normalization of qPCR (Additional file 1: Table S1). qPCR reactions were carried out using 5 µL of master mix (4 µL 2 × KAPA SYBR FAST (Bio-Rad), 0.16 µL 10 µM forward primer, 0.16 µL 10 µM reverse primer, 0.68 µL water per reaction), and 3 µL 1:10 cDNA dilution using a CFX384 thermal cycler (Bio-Rad). Amplification was performed in 384-well plates at 95 °C for 3 min, followed by 43 cycles of 95 °C for 3 s and 60 °C for 30 s, followed by 95 °C for 10 s. To ensure primer specificity, melt curve analyses were carried out at the end of the amplification cycle in increments of 0.5 °C from 65 to 98 °C, each step lasting 5 s. Reactions were carried out in triplicate and relative gene expression was calculated using the ΔΔ-CT method. For statistical comparison of results, two-sided Wilcoxon tests were used as not all data followed a normal distribution.

### Analysis of leaf alkaloid content by LC–MS

Metabolites were extracted from the same leaf powder samples used for qPCR. A volume of 1 mL extraction solvent (60% methanol in water, 0.06% formic acid, 15 mg/L caffeine as internal standard) was added to leaf powder (20–50 mg). The mixtures were shaken vigorously for 2–3 h at room temperature after which they were briefly centrifuged. The clear supernatant was collected and diluted 1:15 with water. Each diluted extract was passed through a 0.22 µm filter and transferred to a glass vial for LC–MS analysis.

LC–MS was conducted on a Thermo Fisher Dionex UltiMate 3000 RS HPLC/UHPLC system coupled to a Bruker compact QqTOF mass spectrometer through an ESI source. The analytes were separated on a Luna C18(2) column (150 × 2 mm, 3 µm, 100 Å, Phenomenex), which was kept at a constant 40 °C. Mobile phases A and B consisted of 0.05% formic acid in water and 0.05% formic acid in acetonitrile, respectively, and the following elution profile was used at a flow rate of 0.3 mL/min: 0–0.5 min, 2% B (constant); 0.5–2.375 min, 2–6% B (linear); 2.375–7 min, 6–25% B (linear), 7–13 min, 25–100% B (linear); 13–14 min, 100% B (constant); 14–14.5 min, 100–2% B (linear); and 14.5–20 min, 2% B (constant). ESI mass spectra were acquired in positive ionization mode with the following parameters: capillary voltage of 4500 V; end plate offset of − 500 V; source temperature of 250 °C; desolvation gas flow of 8.0 L/min; and nebulizer pressure of 2.5 bar. N_2_ was used as desolvation, nebulizer and collision cell gas.

QAs were identified from their exact masses, their UV–vis spectra, and their ESI(+) fragmentations at different collision energies, as in [39]. We could separate two isomers of lupanine and angustifoline with virtually identical UV–vis spectra and mass fragmentation, and we assigned the α-iso-QA structure to the least abundant isomer of the pair [52] (Fig. 3). However, we were unable to fully separate the analogous isomers of 13-hydroxylupanine, and we therefore quantified them as one compound (labelled as “13-hydroxy(iso)lupanine” in Figs. 4, 5 and Additional file 1: Fig. S2). In the case of 13-cinnamoyloxylupanine, we could assign the *cis*- and *trans*-cinnamoyl isomers from their UV–vis spectra, as reported previously [39]. We detected only one isomer of 13-coumaroyloxylupanine, which could be either in *cis* or *trans* configuration (Fig. 3). Similarly, we detected only one isomer of 13-(2-methylbut-2-enoyl)oxylupanine, and this could be either the angeloyl (*cis*) or the tigloyl (*trans*) isomer (Fig. 3). For quantification, alkaloid peaks were normalized to the signal of the internal standard (caffeine) and the fresh weight of the samples. All peak integrations were done on the extracted ion chromatograms of the [M + H]^+^ ions ± 0.005 Da (Additional file 1: Fig. S4). A multivariate analysis of variance (MANOVA) using Pillai’s trace test statistic was used to determine whether QA levels differed significantly between samples. A significant MANOVA (*P* ≤ 0.05) was followed by post-hoc testing of individual QA levels between samples using two-sided Wilcoxon tests, as not all data followed a normal distribution.

## Supplementary Information

**Additional file 1: Fig. S1.** RT-PCR-based amplification of ALSV fragments from systemic *N. benthamiana* leaves infected with ALSV-0 or ALSV-PDS. Expected PCR fragment sizes are indicated above the respective fragments. M, molecular weight marker. The sizes of selected fragments of the molecular weight marker are indicated to their left side (bp). **Fig. S2.** Normalized relative abundance of the ten most abundant QAs in leaves of plants infected with ALSV-GFP or ALSV-PDS. Box plots represent data for 18 or 12 biological replicates for plants infected with ALSV-GFP or ALSV-PDS, respectively. Whiskers represent values within 1.5 times the interquartile range over or under the 75^th^ and 25^th^ percentiles, respectively. Dots and triangles represent the individual data points, the dashed line represents the mean of ALSV-GFP and significant differences are represented by one or two asterisks (two-sided Wilcoxon test; *P* ≤ 0.05 or *P* ≤ 0.01, respectively). **Fig. S3.** Normalized relative expression of *LaPDS* in leaves of plants infected with ALSV–GFP, ALSV–PDS or ALSV–PDS-GFP. Box plots represent data for 5 (ALSV–GFP and ALSV–PDS) or 7 (ALSV–PDS-GFP) biological replicates. Whiskers represent values within 1.5 times the interquartile range over or under the 75^th^ and 25^th^ percentiles, respectively. Dots represent the individual data points, the dashed line represents the mean of ALSV-GFP and the asterisks represent significant differences between leaves of plants infected with ALSV–GFP and ALSV-PDS or ALSV–PDS-GFP (two-sided Wilcoxon test; *P* ≤ 0.01). **Fig. S4.** Representative chromatograms from the LC–MS analysis of leaf extracts from PDS co-silenced plants. (A) Extract of a bleached leaf from an ALSV-PDS-GFP plant (44 mg). (B) Extract of a bleached leaf from an ALSV-PDS-LDC plant (45 mg). The colored, solid traces represent the extracted ion chromatograms (EICs) of the QAs [M + H]^+^ ions ± 0.005 Da. The dashed, light grey traces represent the full-scan base peak chromatograms, superimposed on the EICs. The m/z ranges of the EIC traces are: red, 249.191–249.201; blue, 235.175–235.185; green, 235.175–235.185; violet, 347.228–347.238; light orange, 411.223–411.233; azure, 369.212–369.222; dark grey, 395.228–395.238. The marked peaks are: **1**, 13-hydroxy(iso)lupanine; **2**, isolupanine; **3**, lupanine; **4**, isoangustifoline; **5**, angustifoline; **6**, 13-(2-methylbut-2-enoyl)oxylupanine (angeloyl or tigloyl); **7**, 13-coumaroyloxylupanine (*cis* or *trans*); **8**, 13-benzoyloxylupanine; **9**, 13-*cis*-cinnamoyloxylupanine; **10**, 13-*trans*-cinnamoyloxylupanine; and **11**, caffeine (internal standard). **Sequence S1.** Coding sequence of *LaPDS* from NLL cv. Oskar. The VIGS fragment is highlighted in cyan, and the qPCR amplicon is highlighted in yellow. Individual residues highlighted in grey are located at exon-exon junctions. **Sequence S2.** Coding sequence of *LaLDC* from NLL cv. Oskar. The VIGS fragment is highlighted in cyan, and the qPCR amplicon is highlighted in yellow. *LaLDC* is a single-exon gene. **Sequence S3.** Coding sequence of *LaCAO* from NLL cv. Oskar. The qPCR amplicon is highlighted in yellow. Individual residues highlighted in grey are located at exon-exon junctions. **Sequence S4.** Coding sequence of GFP (U87973). The fragment cloned as a negative control into pALSV-RNA2u is highlighted in cyan. **Table S1**. DNA oligo sequences used in this study.

## Data Availability

The datasets supporting the conclusions of this article are included within the article and its additional files.
