# Supplementary material for: Development and application of a virus-induced gene silencing protocol for the study of gene function in narrow-leafed lupin
Source: Plant Methods. 2021 Dec 28;17:131. doi: 10.1186/s13007-021-00832-4 (PMC8714437; doi:10.1186/s13007-021-00832-4)
Supplement: Supplementary file 1 — Additional file 1: Fig. S1. RT-PCR-based amplification of ALSV fragments from systemic N. benthamiana leaves infected with ALSV-0 or ALSV-PDS. Expected PCR fragment sizes are indicated above the respective fragments. M, molecular weight marker. The sizes of selected fragments of the molecular weight marker are indicated to their left side (bp). Fig. S2. Normalized relative abundance of the ten most abundant QAs in leaves of plants infected with ALSV-GFP or ALSV-PDS. Box plots represent data for 18 or 12 biological replicates for plants infected with ALSV-GFP or ALSV-PDS, respectively. Whiskers represent values within 1.5 times the interquartile range over or under the 75th and 25th percentiles, respectively. Dots and triangles represent the individual data points, the dashed line represents the mean of ALSV-GFP and significant differences are represented by one or two asterisks (two-sided Wilcoxon test; P ≤ 0.05 or P ≤ 0.01, respectively). Fig. S3. Normalized relative expression of LaPDS in leaves of plants infected with ALSV–GFP, ALSV–PDS or ALSV–PDS-GFP. Box plots represent data for 5 (ALSV–GFP and ALSV–PDS) or 7 (ALSV–PDS-GFP) biological replicates. Whiskers represent values within 1.5 times the interquartile range over or under the 75th and 25th percentiles, respectively. Dots represent the individual data points, the dashed line represents the mean of ALSV-GFP and the asterisks represent significant differences between leaves of plants infected with ALSV–GFP and ALSV-PDS or ALSV–PDS-GFP (two-sided Wilcoxon test; P ≤ 0.01). Fig. S4. Representative chromatograms from the LC–MS analysis of leaf extracts from PDS co-silenced plants. (A) Extract of a bleached leaf from an ALSV-PDS-GFP plant (44 mg). (B) Extract of a bleached leaf from an ALSV-PDS-LDC plant (45 mg). The colored, solid traces represent the extracted ion chromatograms (EICs) of the QAs [M + H]+ ions ± 0.005 Da. The dashed, light grey traces represent the full-scan base peak chromatograms, superimposed [file 13007_2021_832_MOESM1_ESM.pdf]

Additional file 1

Development and application of a virus-induced gene silencing protocol for the study of gene function in narrow-leafed lupin

Davide Mancinotti[1†], Maria Cecilia Rodriguez[1†], Karen Michiko Frick[1†], Bjørn Dueholm[1], Ditte Goldschmidt Jepsen[1], Niels Agerbirk[1], and Fernando Geu-Flores[1*]

*Correspondence: feg@plen.ku.dk

†These authors contributed equally to this work

[1]Section for Plant Biochemistry and Copenhagen Plant Science Centre, Department of Plant and Environmental Sciences, University of Copenhagen, Frederiksberg, Denmark

This document contains the following Additional materials:

- Figures S1-S4
- Sequences S1-S4
- Table S1

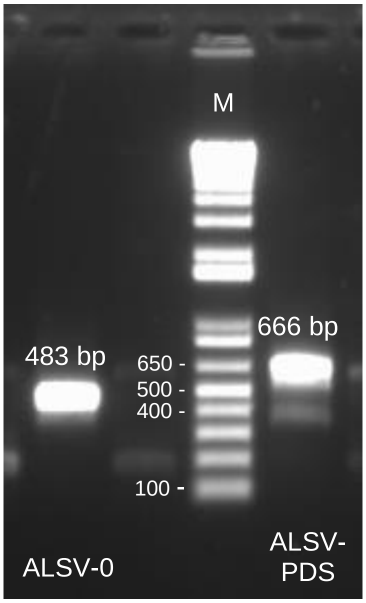

Fig. S1. RT-PCR-based amplification of ALSV fragments from systemic *N. benthamiana* leaves infected with ALSV-0 or ALSV-PDS. Expected PCR fragment sizes are indicated above the respective fragments. M, molecular weight marker. The sizes of selected fragments of the molecular weight marker are indicated to their left side (bp).

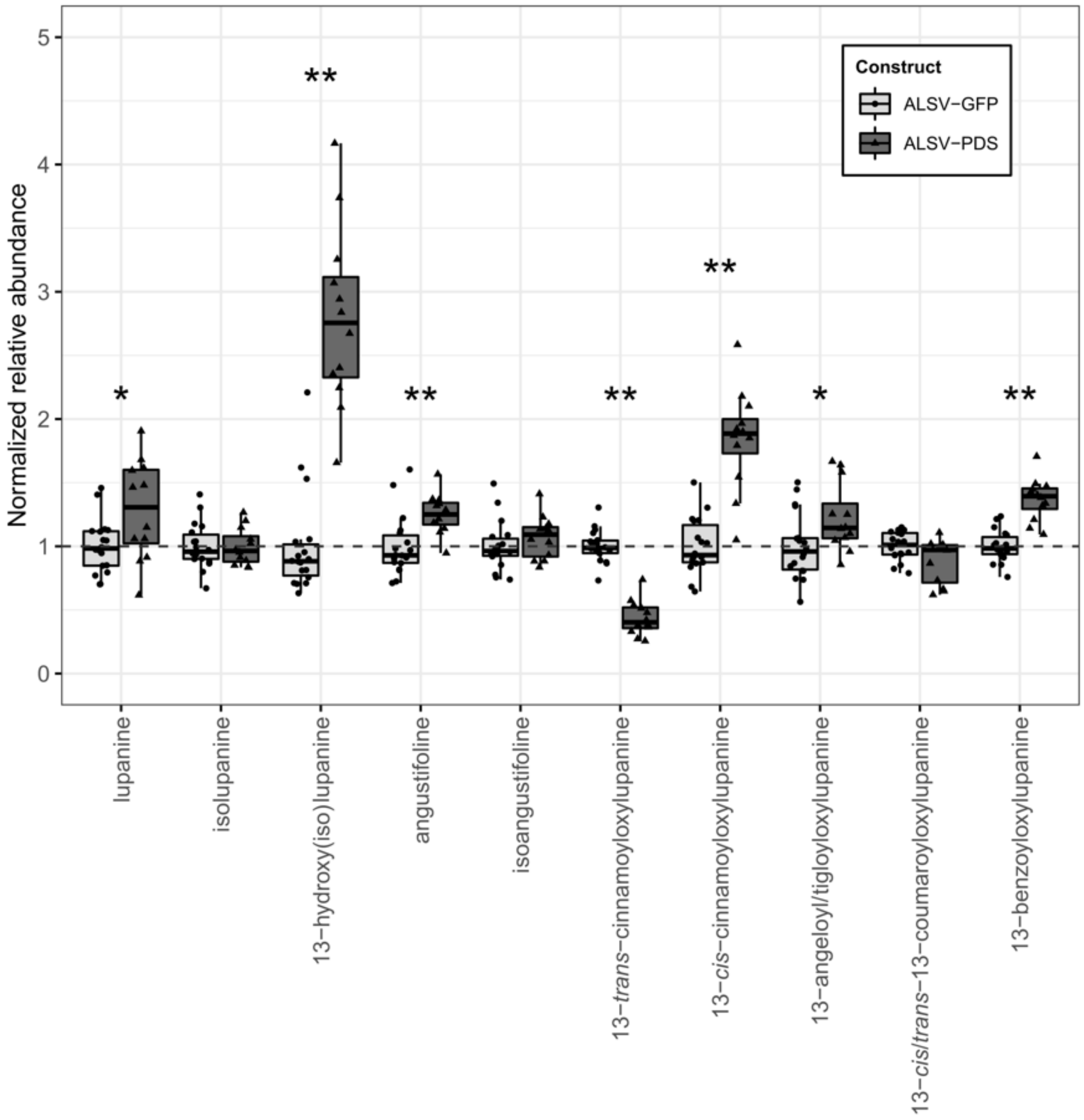

Fig. S2. Normalized relative abundance of the ten most abundant QAs in leaves of plants infected with ALSV-GFP or ALSV-PDS. Box plots represent data for 18 or 12 biological replicates for plants infected with ALSV-GFP or ALSV-PDS, respectively. Whiskers represent values within 1.5 times the interquartile range over or under the 75th and 25th percentiles, respectively. Dots and triangles represent the individual data points, the dashed line represents the mean of ALSV-GFP and significant differences are represented by one or two asterisks (two-sided Wilcoxon test; $P \leq 0.05$ or $P \leq 0.01$, respectively).

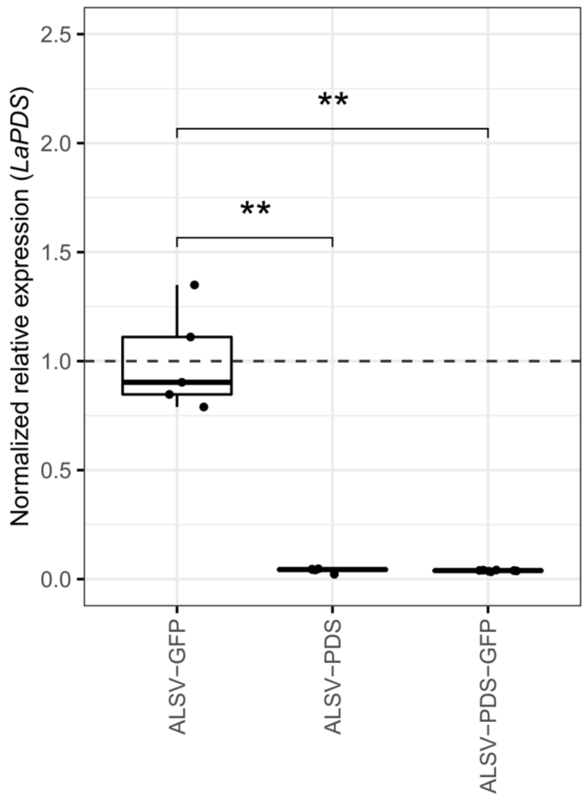

Fig. S3. Normalized relative expression of *LaPDS* in leaves of plants infected with ALSV–GFP, ALSV–PDS or ALSV–PDS-GFP. Box plots represent data for 5 (ALSV–GFP and ALSV–PDS) or 7 (ALSV–PDS-GFP) biological replicates. Whiskers represent values within 1.5 times the interquartile range over or under the 75th and 25th percentiles, respectively. Dots represent the individual data points, the dashed line represents the mean of ALSV-GFP and the asterisks represent significant differences between leaves of plants infected with ALSV–GFP and ALSV-PDS or ALSV–PDS-GFP (two-sided Wilcoxon test; $P \leq 0.01$).

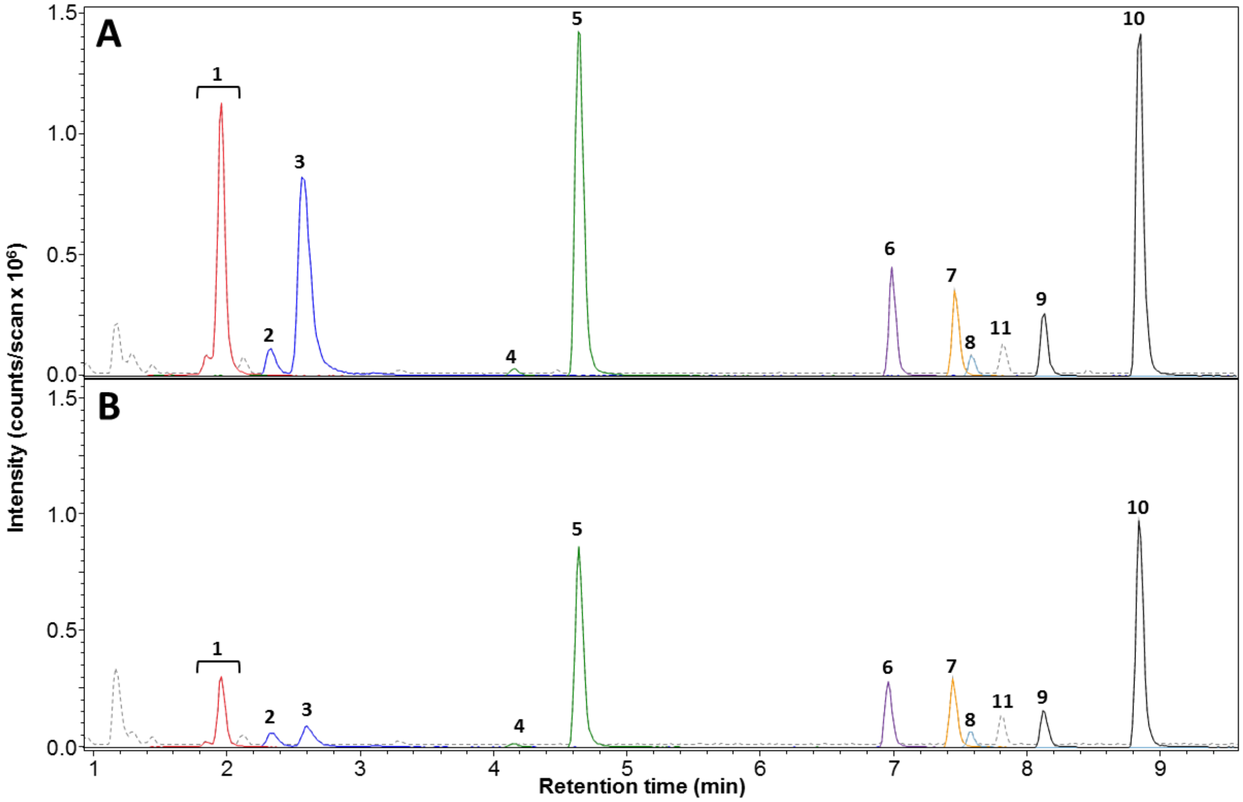

Fig. S4. Representative chromatograms from the LC-MS analysis of leaf extracts from PDS co-silenced plants. (A) Extract of a bleached leaf from an ALSV-PDS-GFP plant (44 mg). (B) Extract of a bleached leaf from an ALSV-PDS-LDC plant (45 mg). The colored, solid traces represent the extracted ion chromatograms (EICs) of the QAs $[M+H]^+$ ions +/- 0.005 Da. The dashed, light grey traces represent the full-scan base peak chromatograms, superimposed on the EICs. The m/z ranges of the EIC traces are: red, 249.191-249.201; blue, 235.175-235.185; green, 235.175-235.185; violet, 347.228-347.238; light orange, 411.223-411.233; azure, 369.212-369.222; dark grey, 395.228-395.238. The marked peaks are: **1**, 13-hydroxy(iso)lupanine; **2**, isolupanine; **3**, lupanine; **4**, isoangustifoline; **5**, angustifoline; **6**, 13-(2-methylbut-2-enoyl)oxylupanine (angeloyl or tigloyl); **7**, 13-coumaroyloxylupanine (*cis* or *trans*); **8**, 13-benzoyloxylupanine; **9**, 13-*cis*-cinnamoyloxylupanine; **10**, 13-*trans*-cinnamoyloxylupanine; and **11**, caffeine (internal standard).

Sequence S1. Coding sequence of *LaPDS* from NLL cv. Oskar. The VIGS fragment is highlighted in cyan, and the qPCR amplicon is highlighted in yellow. Individual residues highlighted in grey are located at exon-exon junctions.

ATGGTTATGGCTTCTTGCATATCTGTGATGAACTTCAATCTCTCAAATGCTTCATCCAAATTC
AATCCAAACCCAATTTCACATCATTCAAAAATCTCATTTTCAACTACCAGATTCTTAAGGAAA
CATCGTTCTCCTCCATTAAGGGTAGTTTGCATGGATTATCCTCGTCCTGAACTTGACAACAC
TGCTAATTTTGTTGAATCTGCTCAATTCTCTTCAACTTTTCGTACTTCTCCACGTCCCACCAA
ACCCTTGAACATAGTTATTGCTGGTGCAGGATTGGCTGGTTTATCAACTGCAAAATATTTGG
CAGATGCTGGTCATAAACCTATTCTGCTGGAGGCAAGAGATGTTCTTGGAGGAAAGATTGC
TGCATGGCAAGATGAAGACGGAGACTGGTATGAGACAGGCCTACATATATTCTTTGGGGCT
TACCCTAACATACAGAACCTGTTTGGAGAACTTGGTATTAATGATCGGTTACAATGGAAGGA
ACATTCCATGATTTTTGCAATGCCAAACAAGCCTGGCGAATTTAGTCGGTTTGATTTTCCGG
ACGTCCTTCCTGCTCCATTAAATGGAATATGGGCAATATTGAAGAACAATGAGATGCTGACT
TGGCCAGAGAAAGTCAAATTTGCAATTGGACTTCTGCCAGCTATGCTTGGCGGGCAGCCTT
ATGTTGAGGCTCAAGATGGCCTTTCTGTGGAAGAATGGATGAAAAAACAGGGCGTTCCTGA
ACGAGTAACTGATGAAGTTTTCATAGCAATGTCAAAGGCACTCAACTTCATCAACCCTGATG
AACTTTCTATGCAATGTATATTGATTGCTTTAAACCGATTTCTTCAGGAGAAGCATGGTTCTA
AGATGGCCTTTTTAGATGGCAATCCCCCCGAAAGACTTTGTATGCCAATTGTTGATCATATT
CAGTCCTTGGGTGGCGAAGTTCATCTCAATTCCCGCATTCAAAAAATTGAGCTAAATGATG
ATGGCACGGTAAAGAACTTCTTACTGAATAATGGCAAAGTGATTGAAGGGGATACTTATGT
GTTTGCAACTCCAGTTGATATTCTGAAGCTTCTTCTGCCTGACAGCTGGAAACAGATTCCAT
ATTTCCAGAGATTAGAGAAATTAGTTGGAGTCCCAGTCATAAACGTTCACATATGGTTTGAC
AGGAAACTGAAGAACACATATGATCACCTTCTCTTTAGCAGAAGTTCCCTTCTGAGTGTATA
TGCTGACATGTCTGTAACTTGTAAGGAATATTATAATCCAAACCAGTCTATGTTGGAGTTAG
TTTTTGCACCAGCTGAAGAATGGATTTCACGTAGCGATGAAGATATTATTGGTGCCACAATG
TCTGAACTTGCTAAACTCTTCCCTGATGAAATTTCTGCTGACCAAAGCAAAGCAAAGATTCT
CAAGTACCATATTGTTAAGACACCAAGGTCGGTTTACAAAACTATTCCAAATTGTGAACCTT
GTCGACCAAGACAAAGATCTCCTATAGAGGGTTTCTATTTAGCTGGAGATTACACAAAACAA
AAATATTTAGCTTCAATGGAAGGTGCTGTTCTTTCTGGGAAGCTTTGTGCACAGGCTATTGT
ACAGGATGCTGAGTTACTTGCTGCTAGGGGCCAAAAAAGGGTGACTCAAGCAGGTGGTGT
TATTTAA

Sequence S2. Coding sequence of *LaLDC* from NLL cv. Oskar. The VIGS fragment is highlighted in cyan, and the qPCR amplicon is highlighted in yellow. *LaLDC* is a single-exon gene.

ATGCCTTCACTACTACTGAGCGAGGGATTATACCAGGCCAAGGGTGCAACAACACCTTTGA
GCCTGAAGGCCATTTATAATGCTTCTGGGGTTAAGGGTAAGCGAGTCACTCCATTACTCGC
TAATGAACAAGAAGGTGGCATCTCTCATTTCATTCAATCCATCATTCGAAACACACCAGATA
TTGATTCACCATTCTTAGTACTTGATCTTGGCGTAATCATGCACCTCATGGAGAAATGGACC
ACTAATCTTCCCACGGTTCAATCTTATTATGCAGTCAAGTGCAACCCTAACCCATCCTTGTT
AGGTGTACTCGCAGCACTTGGTTCGAGCTTCGACTGCGCCAGCCGAGCCGAAATCGAATC
GGTTTTATCACTTGGAGTCTCACCGGACCGGATCATTTATGCGAACCCATGCAAATCAGAG
AGTCACATTAAATATGCAGCTACTGTTGGTGTCAACGTCACAACATTCGATTCTAAAGAAGA
GATCCATAAGATTAAAAAGTGGCACCCAAAATGTGAGTTACTTATTCGTATCAAGCCACCAC
AAGACAGTGGAGCAAGAAATGCTTTGGGTCTCAAATACGGTGCGCTTCCTGAAGAAGTTAA
GCCACTCTTACAAGCCGCTAAAGACGCGGAATTGAAAGTTGTTGGTGTTTCGTTCCACATA
GGAAGTGGTGGTGCTGATTCTAGAACCTACCATGGAGCAATTGCTGCTGCTAAAAGTGTTT
TCGACATGGCTTCTAACGAACTAGGCATGCCAAGAATGAAAATAGTGGACATTGGTGGCG
GTTTCACTTGTGGGAACCAATTTGATGCAGCTTCTTTTCACGTGAATGAGGCTCTTGAGGA
CAATTTTGGAAAAGAGGAAGGTGTTGTGGTAATTGGAGAACCTGGTCGTTATTTTGCTGAG
TCACCTTTTACTTTGGCTAGTAAAGTTATTGGGAAGCGCGTGAGGGGAGAAGTGAGGGAG
TATTGGATTGATGATGGGATTTACGGTTCTCTTAATTGCATAATGTATGACTTTGCAACTGTT
ACTTGCACGCCACTCGCGTGTAGCTCAAAACCAGAGGATCCAGAATGCAAAAACGCCAAA
ATGTACCCTTCAACTGTGTTTGGACCCACTTGTGATTCGTTAGATACTGTTCTAACAGATTA
CTTGTTACCGGAACTGGAACTTGATGATTGGGTTGTGTTCCCAAATATGGGTGCTTATACC
ACATCGTCAGGGACTAATTTCAATGGGTTTAACACAACAGCTATTTCTACTTACCTAGCATA
TTCCACCCCAATTGCGATGGAAAAATCTATGTTCTAA

Sequence S3. Coding sequence of *LaCAO* from NLL cv. Oskar. The qPCR amplicon is highlighted in yellow. Individual residues highlighted in grey are located at exon-exon junctions.

ATGGCTTCAGCTTCTGAAAAAATGGTACCTCCTTCTTGTGCTTGTTGTTCAGCCGGTAATGA
CTCTGCCATCATTCCCCACATTGCGGCTGCCGCCGCTCCCTCTGCTGACTGGACTGCCAA
TGTCCCCGATGATGGCCGCCTAAATAAGATGACCATCGTTCATCCTGTCGACTCCCTACCG
CAACCTTCCATCAATGCCAAAGGAATCATTACACTGCCAAGGCCTCAACCAAGCCACCCTT
TGGACCCTTTATCTCCTGCTGAAATCTCTCTGGCAGTAGCTACTGTGAGGGCTGCTGGAAA
AACTCCTGAGCTTAAAGACGGTTTGCGATTCATGGAAATAGCTTTGCTCGAACCGGATAAA
CATGTCGTTGCACTAGCAGATGCTTATTTTTTTCCACCTTTCCAGCCATCATTGCTTCCTAA
AGGAGGGTTTGTGATCCCAACTAAACTCCCTCCAAGATGTGCTAGACTTCTTGTTTACAATA
GGAAGACAAATGAGACTAGTCTTTGGATCGTCGAGTTATCGCAAGTTCATGCTGTAACTCG
AGGTGGAAATCATTTAGGAAAAGTAATTTCATCACAAGTTGTACCTGATGTTCAGCCTCCAA
TGGATGCTGTGGAGTATGCAGAATGTGAGGCTGCTGTTAAAAGTTATCCTCCATTTATAGA
GGCTATGAAGAAAAGGGGTATTGAAAACATGGAGCTTGTGATGGTAGATCCCTGGTGTGCT
GGTTACTTCAGTGAAGCTGATGATCCGAACCGAAGACTTGCTAAACCAATAATATTTTGCAA
GTGTGAGAGTGATTGCCCTATGGAAAATGGCTATGCTCGCCCGGTCGAGGGAATCTTTGTT
CTTGTTGATATGCAAAAGATGGAGGTGATACAGTTCGAAGACCGCAAACTTGTTCCTCTGC
CTCCTGTAGATCCCTTAAGGAACTATACACATGCTGCAACTAGAGGTGGCACTGATAGAAG
TGACTTAAAACCATTGAAAATTGTTCAACCTGAAGGTCCAAGCTTTTCCGTCAATGGATATT
ATGTTGAATGGCAAAAGTGGAACTTTCGGATTGGATTCACACCCAAAGAAGGTTTAGTTATA
TATTCTGTTGCATATGTTGATGGTAGTCAAGGTCTAAGGCCTGTAGCTCATAGGTTGAGTTT
TGTGGAGATGGTTGTACCCTACGGAGATCCAAACGATCCACATTACAGGAAAAATGCTTTT
GATGCTGGGGAAGATGGCCTAGGAAGAAATGCACATTCCTTGAAGAAGGGATGTGATTGT
TCTGGCATAGTCAAATATTTTGATGCTCACTTCACAAATTTCACTGGTGGTGTGGAGACAAT
TGAAAATTGTGTATGTTTGCATGAAGAAGATCATGGAATTCTTTGGAAGCATCAAGATTGGA
GAACTGGCTTATCAGAAGTCCGAAGGTCTAGAAGGCTTTCAGTTTCATTTATATGTACTGTG
GCTAACTATGAGTATGGATTTTTTTGGCACTTTTATCAGGATGGAAAGATGGAAGCTGAAGT
TAAGCTAACTGGAATTCTGAGCATGGGAGCCTTAATGCCCGGAGAGTATCGAAAATATGGA
ACCGTGATTGCCCCAGGTCTATATGCTCCAGTTCATCAACACTTTTTTGTTGCTCGTATGAA
CATGGCTGTTGATTCTAGACCTGGTGAAGCTTTGAATCAGGTTGTGGAAGTCAATGTGAAA
GCTGAGGAACCTGGTGATCATAATGTTCACAATAATGCATTCTATGCCGAAGAAACTTTGCT
CAGATCTGAAATGGAAGCAATGCGTGATTGCGATCCCATGACTGCTCGATCTTGGATTGTA
AGGAATACAAGATCAACCAATAGAACTGGACACTTGACAGGCTACAAGCTAGTACCTGGCT

CGAACTGCTTACCATTCGCGCATTCGGATGCCAAGTTTTTAAGAAGAGGTGCTTTCTTGAA
GCATAATCTTTGGGTTACAGCTTACTCACCCGATGAGCTGTTTCCTGGAGGAGAATTTCCT
AATCAAAATCCACGCATTGGCGACGGATTACCTACATGGGTTACGCAGAACCGATCTTTAG
AAGAGTCTGATATAGTTCTTTGGTATGTATTTGGAGTCACACATGTTCCTCGTTTAGAAGAC
TGGCCTGTTATGCCAGTAGAGCACATTGGTTTTATGCTCATGCCTCATGGATTCTTCAATTG
TTCCCCTGCAATAGATGTTCCACCTAGTAAATGTGAATTGGAGGCTAAAGAAAAAGATATAA
AGGATAATGGGGTTTTGAAGCCAATTGAGAATTCCTTAGCATCAAAGCTCTAA

Sequence S4. Coding sequence of GFP (U87973). The fragment cloned as a negative control into pALSV-RNA2u is highlighted in cyan.

ATGAGTAAAGGAGAAGAACTTTTCACTGGAGTTGTCCCAATTCTTGTTGAATTAGATGGTGA
TGTTAATGGGCACAAATTTTCTGTCAGTGGAGAGGGTGAAGGTGATGCAACATACGGAAAA
CTTACCCTTAAATTTATTTGCACTACTGGAAAACTACCTGTTCCATGGCCAACACTTGTCAC
TACTTTCTCTTATGGTGTTCAATGCTTTTCAAGATACCCAGATCATATGAAGCGGCACGACT
TCTTCAAGAGCGCCATGCCTGAGGGATACGTGCAGGAGAGGACCATCTTCTTCAAGGACG
ACGGGAACTACAAGACACGTGCTGAAGTCAAGTTTGAGGGAGACACCCTCGTCAACAGGA
TCGAGCTTAAGGGAATCGATTTCAAGGAGGACGGAAACATCCTCGGCCACAAGTTGGAAT
ACAACTACAACTCCCACAACGTATACATCATGGCCGACAAGCAAAAGAACGGCATCAAAGC
CAACTTCAAGACCCGCCACAACATCGAAGACGGCGGCGTGCAACTCGCTGATCATTATCA
ACAAAATACTCCAATTGGCGATGGCCCTGTCCTTTTACCAGACAACCATTACCTGTCCACA
CAATCTGCCCTTTCGAAAGATCCCAACGAAAAGAGAGACCACATGGTCCTTCTTGAGTTTG
TAACAGCTGCTGGGATTACACATGGCATGGATGAACTATACAAATAA

Table S1. DNA oligo sequences used in this study.

Name	Sequence (5' to 3')	Purpose
ALSV2_USER(+)	Phos-TCGAGGCTGAGGCCTTAATTAACCTCAGCG	Insert a USER cassette into pEALSR2L5R5
ALSV2_USER(-)	Phos-GATCCGCTGAGGTTAATTAAGGCCTCAGCC	Insert a USER cassette into pEALSR2L5R5
ALSV_35S_Fw	GGCTTAAUCCAGTGCCAAGCTTGCC	Generate pALSV-RNA1u and pALSV-RNA2u
ALSV1_Frag1_Rv	ACCCTTGCCCUCTGAGAGCTT	Generate pALSV-RNA1u
ALSV1_Frag2_Fw	AGGGCAAGGGUTGCAATGGTG	Generate pALSV-RNA1u
ALSV1_Frag2_Rv	AAAGAACUATTCTCTAAAAAATAAGGC	Generate pALSV-RNA1u
ALSV1_Frag3_Fw	AGTTCTTUAACTCCCTCTCTAATTCATG	Generate pALSV-RNA1u
ALSV2_Frag1_Rv	ATTCTGGAGUAGAAGAAAAGAAAATTTTCCCTT	Generate pALSV-RNA2u
ALSV2_Frag2_Fw	ACTCCAGAAUTTACCAGGGAGGACCTTGTT	Generate pALSV-RNA2u
ALSV_Tnos_Rv	GGTTTAAUGAATTCCCGATCTAGTAACATAGA	Generate pALSV-RNA1u and pALSV-RNA2u
pALSV-RNA2u_Fw	GAGAAAGATCGGAATGAAG	Verify pALSV-RNA2u insert (sequencing) and viral infection (RT-PCR)
pALSV-RNA2u_Rv	TTGGGGAGGGCAAATGGAAA	Verify pALSV-RNA2u insert (sequencing) and viral infection (RT-PCR)
PDS_pALSV-RNA2u_Fw	GGCCTTAAUATGGATTTCACGTAGCGATG	Clone *LaPDS* fragment into pALSV-RNA2u
PDS_pALSV-RNA2u_Rv	GGTTAAUTTGTCTTGGTCGACAAGGTT	Clone *LaPDS* fragment into pALSV-RNA2u
GFP_pALSV-RNA2u_Fw	GGCCTTAAUAGACACCCTCGTCAACAGG	Clone GFP fragment into pALSV-RNA2u
GFP_pALSV-RNA2u_Rv	GGTTAAUGCCGCCGTCTTCGATGTTG	Clone GFP fragment into pALSV-RNA2u
LDC_pALSV-RNA2u_Fw	GGCCTTAAUTAGTAAAGTTATTGGGAAGCG	Clone *LaLDC* fragment into pALSV-RNA2u
LDC_pALSV-RNA2u_Rv	GGTTAAUAGTGGGTCCAAACACAGTTGAA	Clone *LaLDC* fragment into pALSV-RNA2u
PDS_3'Fus_Rv	AGATCTTUGTCTTGGTCGACAAGGTTCACAA	Clone *LaPDS* fragment as 5' gene fusion into pALSV-RNA2u (in combination with primer PDS_pALSV-RNA2u_Fw)
GFP_5'Fus_Fw	AAAGATCUGACACCCTCGTCAACAGGATC	Clone GFP fragment as 3' gene fusion into pALSV-RNA2u (in combination with primer GFP_pALSV-RNA2u_Rv)
LDC_5'Fus_Fw	AAAGATCUAGTAAAGTTATTGGGAAGCGCGT	Clone *LaLDC* fragment as 3' gene fusion into pALSV-RNA2u (in combination with primer LDC_pALSV-RNAu_Rv)
Ubi_qPCR_Fw	TGACAGCCCACTGAATTGTGAT	Reference gene for qPCR (Lup024215)
Ubi_qPCR_Rv	TCTTGGGCATAGCAGCAAGC	Reference gene for qPCR (Lup024215)
PDS_qPCR_Fw	TCCCAGTCATAAACGTTC	Quantify *LaPDS* transcript via qPCR (fwd primer)
PDS_qPCR_Rv	CTGGTGCAAAAACTAACTC	Quantify *LaPDS* transcript via qPCR (rev primer)
LDC_qPCR_Fw	ACCGGAACTGGAACTTGATG	Quantify *LaLDC* transcript via qPCR (fwd primer)
LDC_qPCR_Rv	TTGGGGTGGAATATGCTAGG	Quantify *LaLDC* transcript via qPCR (rev primer)
CAO_qPCR_Fw	ACTCACCCGATGAGCTGTTTC	Quantify *LaCAO* transcript via qPCR (fwd primer)
CAO_qPCR_Rv	GGCCAGTCTTCTAAACGAGG	Quantify *LaCAO* transcript via qPCR (rev primer)
